# Analysis of the transplacental transmission of SARS CoV-2 virus and antibody transfer according to the gestational age at maternal infection

**DOI:** 10.1038/s41598-024-53580-5

**Published:** 2024-02-11

**Authors:** Louise Lucot-Royer, Camille Nallet, Manon Vouga, Marc Puyraveau, Frederic Mauny, Solène Marty-Quinternet, Charline Bertholdt, Jean-Paul Bory, Christine Devalland, Margaux Canaguier, Camille Copolla, Marie-Laure Eszto, Yohny Montoya, Marion Roesch, Sandrine Reviron, Didier Riethmuller, Emma Rufenacht, Emmanuel Simon, Nicolas Mottet

**Affiliations:** 1 Pôle Mère-Femme, Department of Obstetrics and Gynecology, University Hospital of Besancon, University of Franche-Comte, Alexander Fleming Boulevard, 25000 Besançon, France; 2 Nanomedicine Lab, Imagery and Therapeutics, EA4662, University of Franche-Comte, 25000 Besançoon, France; 3 Department of Obstetrics and Gynecology, University Hospital of Nancy, Nancy Hopital Central, 54000 Nancy, France; 4 Department of Obstetrics and Gynecology, University Hospital of Reims, 51092 Reims, France; 5 Department of Obstetrics and Gynecology, Hopital Nord Franche Comté, 90400 Trévenans, France; 6 Department of Obstetrics and Gynecology, Groupe Hospitalier de la Haute-Saone, 70000 Vesoul, France; 7 Department of Obstetrics and Gynecology, CHR Metz-Thionville, 57100 Thionville, France; 8 Department of Obstetrics and Gynecology, Hopital Jura Sud, 39000 Lons-Le-Saunier, France; 9 Department of Obstetrics and Gynecology, University Hospital of Grenoble, CHU Grenoble Alpes, 38700 La Tronche, France; 10 Department of Obstetrics and Gynecology, Centre Hospitalier Intercommunal de Haute Comté, 25300 Pontarlier, France; 11 Department of Obstetrics and Gynecology, University Hospital of Dijon, CHU Mitterand, 21000 Dijon, France

**Keywords:** Occupational health, Epidemiology, Outcomes research, Infection, Viral infection

## Abstract

To quantify transplacental transmission of SARS-CoV-2 virus and antibody transfer in pregnant women and their newborns according to the gestational age at maternal infection. A prospective observational multicenter study including pregnant women with a positive RT-PCR or a positive serology for SARS-CoV-2 and compatible symptoms, from April to December 2020, in 11 French maternities. The study was designed to obtain a systematic collection of mother-infant dyad’s samples at birth. SARS-CoV-2 viral load was measured by RT-PCR. IgG and IgM antibodies against the SARS-CoV-2 spike protein were measured by enzyme-linked immunosorbent assay. Antibody concentrations and transplacental transfer ratios were analyzed according to the gestational age at maternal infection. The primary outcome was the rate of SARS CoV-2 materno-fetal transmission at birth. The secondary outcome was the quantification of materno-fetal antibody transfer. Maternal and neonatal outcomes at birth were additionally assessed. Among 165 dyads enrolled, one congenital infection was confirmed {n = 1 (0.63%) IC_95%_ [0.02%; 3.48%]}. The average placental IgG antibody transfer ratio was 1.27 (IC 95% [0.69–2.89]). The transfer ratio increased with increasing time between the onset of maternal infection and delivery (*P* Value = 0.0001). Maternal and neonatal outcomes were reassuring. We confirmed the very low rate of SARS-CoV-2 transplacental transmission (< 1%). Maternal antibody transfer to the fetus was more efficient when the infection occurred during the first and second trimester of pregnancy.

## Introduction

At the beginning of the Severe Acute Respiratory Syndrome Coronavirus 2 (SARS-CoV-2) pandemic, it was unclear whether SARS-CoV-2 could be transmitted from the mother to the fetus. Cases of perinatal transmission were described, without knowing whether these occurred through transplacental transmission or through environmental exposure at birth. Vivanti et al.^1^ demonstrated, in July 2020, the transplacental transmission of SARS-CoV-2 in a neonate born to a mother infected during the third trimester. Very early during the pandemic the expression of the ACE2 receptor, known to play an important role in SARS-CoV-2 replication, was described in the placenta, raising fears of a possible viral replication in the placenta^2–7^. Face with the urgent need to have data regarding this unexpected crisis, the number of publications exploded with many heterogeneities in the clinical presentation of pregnant women and gestational age at infection making it difficult to interpret the risk of vertical transmission.

Today, little is known about the effect of SARS CoV-2 on pregnancy depending on the gestational age at maternal infection and growing evidence suggests that the susceptibility of the human placenta and, perhaps, the fetal tissues to the infection may vary depending on the gestational age^8–14^. On the other hand, maternal IgG antibodies are able to cross the placenta and could potentially offer a protection to fetus and the newborn against SARS-CoV-2 infection. Accurate evaluation of materno-fetal transmission during pregnancy and quantification of placental antibody transfer remain controversial because, in most of the studies, a systematic analysis of materno-fetal transmission was lacking^15–19^. A recent review by Kotlyar et al*.* suggested that materno-fetal transmission of the virus could reach 3.2% in mothers infected during the third trimester. However, fetal infection could only be convincingly determined by the direct demonstration of the presence of SARS-CoV-2 in amniotic fluid or detection of the virus by PCR in umbilical cord blood/neonatal blood collected within first 12 h of birth (classification of Prakesch^20^) in a minority of cases. Regarding antibody transfer, results are still discordant^21–25^. Although, Flannery et al*.*^26^ detected specific IgG in 86.7% (72/83) of exposed fetuses, information regarding the potential impact of the delay between maternal infection and delivery regarding materno-fetal antibody transfer is still being explored^27–31^. Due to the significant protection offered by passive immunization acquired through maternal antibodies to the vulnerable newborn and its potential impact on vaccination campaigns, comparing the efficiency of placental antibody transfer in the first, second, and third trimester of infection with SARS-CoV-2 needs to continue to be explored.

The TRANSCOVID Study aimed to quantify transplacental transmission of SARS-CoV-2 virus and antibody transfer in pregnant women and their newborns according to the gestational age at maternal infection. None of the data from the study has already been published.

## Methods

### Study design and population

We conducted a prospective observational descriptive longitudinal study on pregnant women with a confirmed SARS-COV-2 infection. Women were approached prospectively for enrollment in 11 obstetrics care centers (Minjoz-Besancon, Dole, Lons-Le-Saunier, Vesoul, Pontarlier, Nord-Franche-Comte, Dijon, Grenoble, Mulhouse, Nancy, Thionville, and Reims hospitals) from April to December 2020. During this period, pregnant women were tested for SARS-CoV-2 by nasopharyngeal reverse transcription polymerase chain reaction (NP-RT-PCR) and/or serology after obtaining their consent in two settings: (1) routinely, upon admission to the hospital for delivery if they had presented any symptoms compatible with a SARS-CoV-2 infection during pregnancy, and (2) at any gestational age during pregnancy if they presented compatible symptoms or a potential SARS-CoV-2 exposure. Pregnant women were eligible for inclusion if they were aged 18 years or older, able to provide informed consent or had a health care proxy able to do so and diagnosed with a confirmed SARS-CoV-2 infection. The TRANSCOVID Study was approved by institutional review boards CPP 8 Ile de France (ID-RCB 2020-A01066-33, N° CPP: 20 04 12), registered on clinical trial (NCT04402918). The study protocol was performed according to the Declaration of Helsinki principles, and the written informed consent containing the details of the study was obtained from all participants.

### Diagnosis of maternal SARS CoV-2 infection

**Figure 1:**
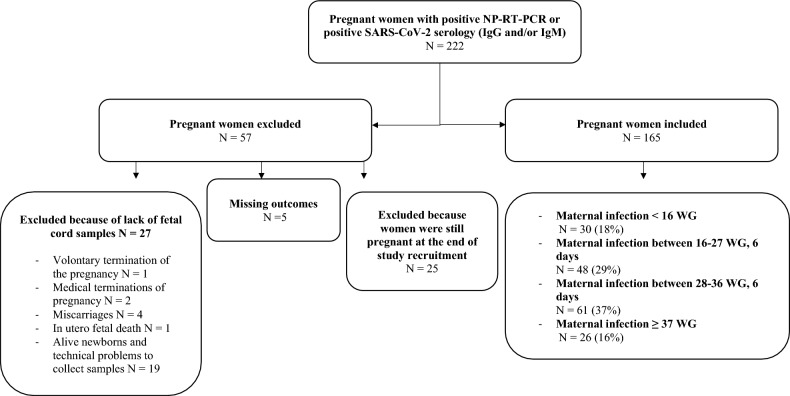
Flowchart.

Confirmed maternal SARS-CoV-2 infection was defined as a positive RT-PCR for SARS-CoV-2 or a positive serology defined as the presence of either specific IgM and/or specific IgG with a history of maternal symptoms (Fig. 1). Timing of maternal infection diagnosed by a positive serology was determined based on the timing of maternal symptoms. As the study was performed before the availability of vaccination, a positive serology was considered as a definite past exposition to SARS CoV-2. Included women were classified according to the gestational age at maternal infection: before 16 weeks of gestation (WG), from 16 to 28 WG and from 28 to 42 WG. The third trimester was deliberately split into two categories: before and after 37 WG in order to distinguish SARS-CoV-2 infections that occurred very close to delivery.

### Definition of outcomes

**Figure 2:**
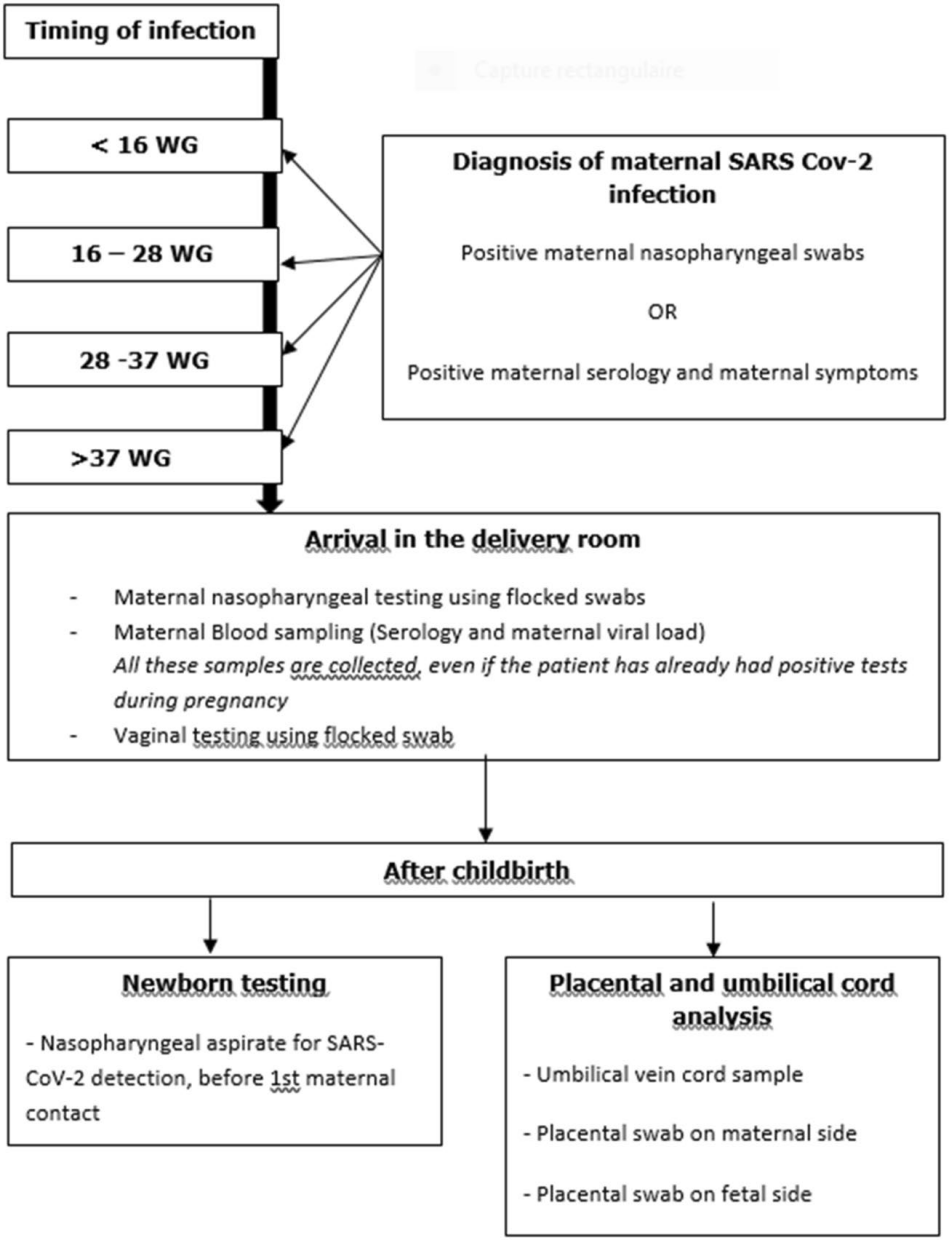
Diagnosis of maternal infection and collected samples.

The primary outcome was the rate of materno-fetal infection. Type of materno-fetal infection ((confirmed, probable, possible, unlikely or not infected) was defined according to Shah et al*.*^20^ classification based on clinical features and results of the biological samples at delivery. The secondary objective was the evaluation of materno-fetal IgG antibody transfer assessed by a transfer ratio defined as the neonatal cord blood IgG concentration/maternal serum IgG concentration. Nasopharyngeal (NP) sampling using flocked swabs and serology testing were used to characterize maternal and fetal/neonatal SARS-CoV-2 infection at the time of delivery. Maternal NP was repeated at delivery if the infection had been diagnosed more than one week before the delivery to assess the persistence of SARS-CoV-2 replication. Upon admission to delivery room, maternal blood samples were systematically collected for SARS-CoV-2 RT-PCR and serology testing. In case of abortion, the same biological samples were collected. Swabs were stored at 4 °C when processed within 24h or stored at − 80°C when processed thereafter. A vaginal swab was collected at admission with sterile nylon flocked swabs. To diagnose a congenital infection, a swab was collected from the oropharynx and trachea (if the newborn was intubated) immediately after birth. Umbilical blood samples (5–10 ml) were collected by needle puncture after cord clamping with sterile gloves to limit risk of contamination. The serum and plasma were separated and aliquoted. All samples were stored at − 20 °C until analysis. Placental swabs (both maternal and fetal sides) were obtained with sterile nylon flocked swabs and were stored at 4 °C during 24h or at − 80 °C when processed thereafter (Fig. 2).

Viral RNA was extracted using the NucleoMag Pathogen Kit (Macherey–Nagel) on Nimbus Presto platform (Hamilton) according to the manufacturer’s instructions and amplified by RT-PCR using a protocol developed by Charite (E gene) and Institut Pasteur (RdRp gene) as previously described^32^ from March to June 2020 or by the TaqPath COVID-19 CE-IVD RT-PCR Kit (Thermofisher) since June 2020. Results were obtained in cycle threshold (Ct).

Serological assays were performed using the ELISA Anti-SARS-CoV-2 QuantiVac (IgG) and the ELISA anti-SARS-CoV-2-NCP IgM kits (Euroimmun) on EVOLIS system (Bio-Rad), according to the manufacturer’s instructions, for IgG and IgM detection, respectively. IgG were directly quantified and expressed in BAU/ml with a positive threshold at 25.6 BAU/ml, according to the manufacturer’s instruction. Information on patients’ demographics and history as well as details of treatments and results from exams were recorded in an electronical database (CleanWeb^®^).

### Statistical analysis

We evaluated the number of patients to include in the cohort to 160 based on a cumulative annual number of deliveries estimated in the participating centers of 29,000 during the study period, a contamination rate in the target population between 5 and 15%, associated with proportion of symptomatic infections of 15%, the realization of a nasopharyngeal swab of 95% in these symptomatic pregnant patients and a sensitivity of the RT-PCR on nasopharyngeal swab of 70%. Applying a 5% loss of unanalyzable data, we expected 150 women to be included in the study. In case of absence of viral transmission (expected proportion equal to zero), the 95% confidence interval of the proportion of children infected with SARS Cov-2 based on a Poisson distribution would be [0; 2.5%], for a 1% viral transmission it would be [0.1; 4.3] and finally for a 5% viral transmission it would be [2.1; 10.1].

Descriptive statistics are presented as means and standard deviations (SD) or as frequencies and percentages (%). Correlations between maternal and infant IgG concentrations and transfer ratio and days between gestational age at maternal infection and delivery were reported using the Pearson correlation coefficient. We used Student tests to explore the transfer ratio function of the gestational age at maternal SARS-CoV-2 infection at.

### Ethical approval

Réf CPP: 20 04 12. Réf CNRIPH: 20.04.16.57739. Réf. Protocole: 2020/500—Etude TRANSCOVID. N° ID RCB: 2020-A01066-33. Date: 30th April 2020.

## Results

During the study period, 222 pregnant women were included, and matched maternal-cord blood sera were finally available for 165 mother-infant dyads, including 3 twin pregnancies (Fig. 2). 57 patients were excluded from the final analysis. 30 (18%) pregnant women were infected < 16 WG, 48 (29%) between 16 and 27 WG and 6 days, 61 (37%) between 28 and 36 WG and 6 days and 26 (16%) ≥ 37 WG. The characteristics of the 165 mother-infant dyads as well as the neonatal outcomes are presented in Table 1. Among the 165 dyads analyzed, SARS-CoV-2 RNA was amplified from 5 placentas on maternal side, 6 placentas on the fetal side and one vaginal sample (Table 2).

**Table 1:** Characteristics of the mother/newborn dyads.

Gestational age at maternal infection Week of gestation	[0–16] N = 30	[16–28] N = 48	[28–37] N = 61	[37–42] N = 26	*p* value
Age Mean (SD)	32.5 (± 5.11)	31.20 (± 4.01)	31.75 ± (4.89)	30.31 (± 5.12)	0.3257^a^
BMI Mean (SD)	25.14 (± 6.44)	25.88 (± 5.85)	26.29 (± 5.05)	24.65 (± 3.89)	0.5631^a^
Gestational age at maternal infection Mean (SD)	9.77 (± 3.85)	23.52 (± 3.78)	34.21 (± 2.39)	38.96 (± 0.92)	< 0.001^a^
Symptomatic infection N (%)	16 (53%)	31 (64.5%)	32 (52. 4%)	11 (42.3%)	0.1259^b^
Hospitalization N (%)	1 (3.3%)	1 (2.0%)	6 (9.8%)	4 (15.4%)	0.0009^b^
Comorbidity N (%)	6 (20%)	11 (22.9%)	15 (24.6%)	4 (15.4%)	0.8018^b^
Hypertension N (%)	0	2 (4.2%)	4 (6.6%)	1 (3.9%)	0.06109^b^
Diabetes N (%)	5 (16.7%)	5 (10.4%)	9 (14.8%)	3 (11.5%)	0.8454^b^
Smoking N (%)	4 (13.3%)	1 (2.1%)	1 (1.6%)	3 (12%)	0.0294^b^
Gestational age at delivery Mean (SD)	39.03 (± 1.30)	38.74 (± 2.64)	38.67 (± 1.95)	39.92 (± 1.06)	0.0488^a^
C-Section N (%) Vaginal delivery N (%)	4 (13.3%) 26 (86.7%)	7 (14.6%) 41 (85.4%)	10 (16.4%) 51 (83.6%)	7 (26.9%) 19 (73.1%)	0.5091^b^
Weight at birth Mean (SD)	3310.17 (± 461.05)	3148.21 (± 661.71)	3296.69 (± 664.03)	3398.85 (± 467.57)	0.3388^a^
NICU transfer N (%)	1 (3.3%)	5 (10.4%)	8 (13.1%)	0	0.1489^b^
Prematurity N (%)	1 (3.3%)	5 (10.4%)	5 (8.2%)	0	0.3583^b^
IUGR N (%)	1 (3.3%)	0	2 (3.3%)	0	0.5638^b^

Data are expressed as number (percentage) or as mean (standard derivation). Age (years), BMI (Kg/cm^2^), Date (WG).

*IUGR* Intra uterine growth restriction.

^a^ANOVA.

^b^Chi^2^.

**Table 2:** Maternal and neonatal samples results at delivery according to the gestational age of maternal infection.

Gestational age at maternal infection (Week of gestation)	[0–16] N = 30	[16–28] N = 48	[28–37] N = 61	[37–42] N = 26	*p* value
Maternal nasopharyngeal SARS-COV2* RT-PCR					< 0.0001^b^
Negative	23 (95.8%)	26 (81.3%)	38 (70.4%)	7 (33.3%)	
Positive	1 (4.2%)	6 (18.7%)	16 (29.6%)	14 (66.7¨%)	
Vaginal SARS-COV2 RT-PCR*					1^b^
Negative	27 (100%)	42 (100%)	50 (98.0%)	21 (100%)	
Positive	0	0	1 (2.0%)	0	
Placental SARS-COV2 RT-PCR maternal pole *					0.6958^b^
Negative	28 (100%)	44 (97.8%)	52 (94.6%)	22 (95.7%)	
Positive	0	1 (2.2%)	3 (5.4%)	1 (4.3%)	
Maternal antibodies level (BAU/mL) †					
IgG	54.0 (± 48.9)	58.8 (± 75.5)	112.4 (± 138.8)	44.0 (± 75.2)	
IgM	0.28 (± 0.23)	0.41 (± 0.48)	1.24 (± 2.24)	0.95 (± 2.10)	
Placental SARS-COV2 RT-PCR fetal pole *					0.3739^b^
Négative	28 (100%)	44 (97.8%)	52 (94.6%)	22 (91.7%)	
Positive	0	1 (2.2%)	3 (5.4%)	2 (8.3%)	
Fetal nasopharyngeal SARS-COV2 RT-PCR *					
Negative	28 (100%)	39 (100%)	53 (100%)	24 (100%)	
Positive	0	0	0	0	
blood cord SARS-COV2 RT-PCR *					
Negative	29 (100%)	46 (100%)	56 (98.3%)	25 (100%)	
Positive	0	0	1 (1.7%)	0	
Fetal serology †					
IgG	80.6 (± 69.9)	79.4 (± 88.9)	96.4 (± 115.8)	26.0 (± 49.9)	
IgM	0.05 (± 0.03)	0.07 (± 0.09)	0.08 (± 0.07)	0.08 (± 0.09)	

*Data are expressed as number (percentage).

^†^Data are expressed as mean (standard derivation).

^b^Chi^2^.

Missing data (N = n): NP-RT-PCR (N = 35), Vaginal PCR (N = 25), Placental RT- PCR maternal (N = 15), Placental RT-PCR fetal (N = 14) Fetal RT-PCR (N = 22) RT-PCR blood cord (N = 9).

One confirmed congenital infection was observed {(0.63%) IC_95%_ [0.02%; 3.48%]}, none were probable, none were possible. The confirmed infection was reported in a 34-year-old patient who tested positive for SARS-CoV-2 at 37 WG and 2 days. The patient was Caucasian with a normal pregnancy. The patient was asymptomatic and initial diagnosis was made due to close contact with an infected person. She presented at 37 WG and 4 days for reduced fetal movements. During follow-up, fetal monitoring was abnormal with loss of variability and decelerations. The medical team therefore performed a caesarean section before labor for a non-reassuring fetal status. The patient gave birth to a eutrophic female newborn for the gestational age, weighing 2760 g with an Apgar score 7–10–10. The arterial cord blood pH was 7.28. The newborn's condition did not require transfer to neonatal intensive care. All the maternal, newborn and placental swabs were positive for SARS-CoV-2. Following delivery, both maternal and neonatal course were uneventful.

**Figure 3:**
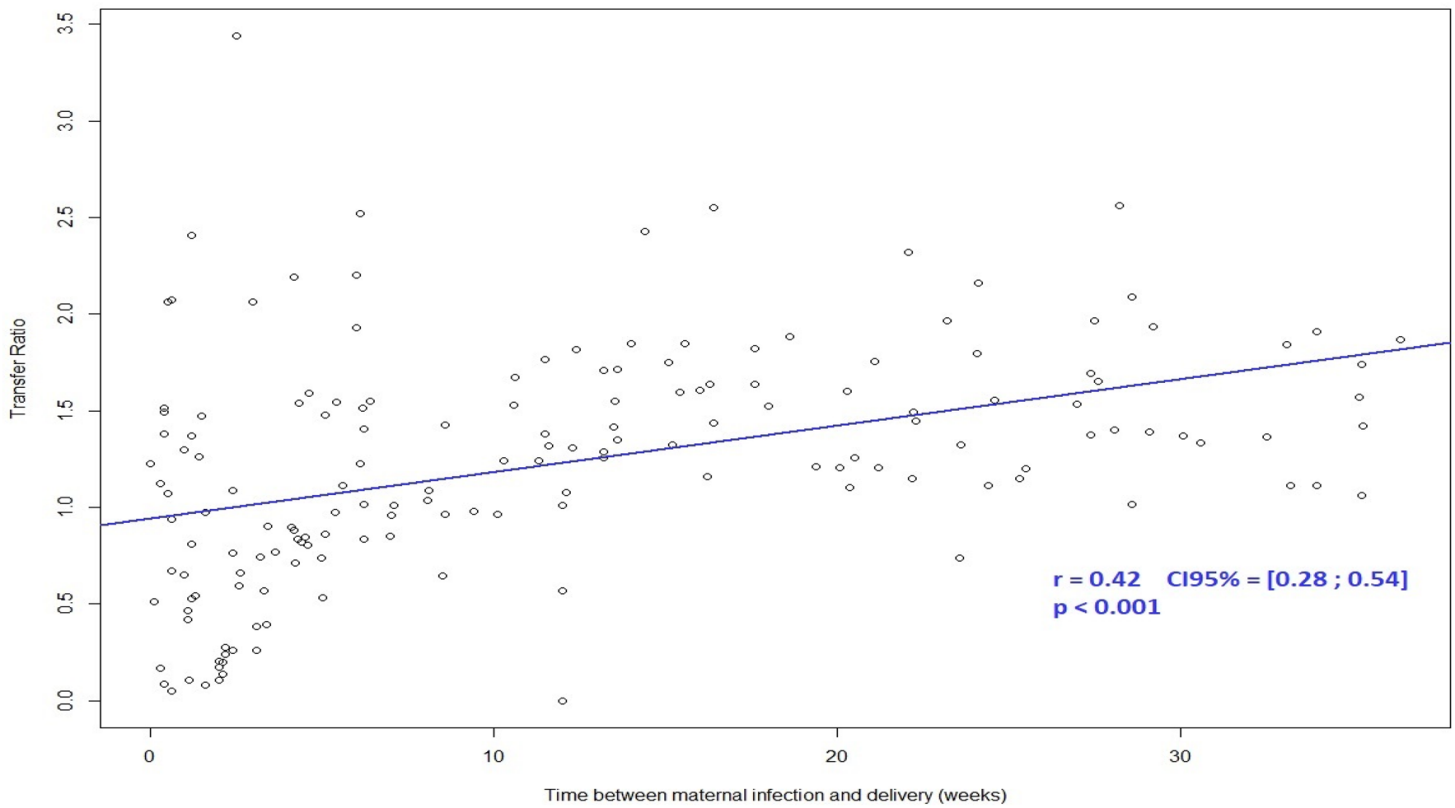
Transfer ratio according to time between infection and delivery.

Among 165 maternal sera tested, 106 (64.2%) were positive for specific SARS-CoV-2 IgG. The average placental antibody transfer ratio was 1.27 (IC 95% [38]). Transfer ratios did not differ between infants born to mothers with an asymptomatic or a symptomatic infection (*p* value = 0.4805) (Table 3). The transfer ratio increased with increasing time between the onset of maternal infection and delivery (*P* Value = 0.0001) (Table 3, Fig. 3) and placental antibody transfer seemed to be more efficient after a maternal infection during the first trimester. Results of maternal and neonatal IgG and IgM concentration are presented in Table 2.

**Table 3:** Transfer ratio according to the gestational age of infection at SARS-CoV-2 or the symptomatic statut.

	N	Mean (SD)	IC95%	Minimum	Median	Maximum	*p* value
Global population	106	1.27 (± 0.57)	[1.16–1.38]	0.05	1.32	3.44	
Symptomatic population							0.4805a
Yes	32	1.34 (± 0.69)	[1.09–1.58]	0.11	1.38	3.44	
No	66	1.25 (± 0.54)	[1.11–1.38]	0.05	1.28	2.52	
Infection Week of gestation							< 0.0004b
[0–16]	23	1.55 (± 0.33)	[1.41–1.69]	1.01	1.42	2.16	
[16–28]	33	1.44 (± 0.44)	[1.28–1.59]	0.08	1.44	2.43	
[28–37]	42	1.07 (± 0.64)	[0.87–1.27]	0.11	0.90	3.44	
[37–42]	8	0.88 (± 0.71)	[0.28–1.47]	0.05	0.91	2.061	

^a^Student test comparing the mean transfer ration between the asymptomatic group and the symptomatic group.

^b^Analysis of variance comparing the mean transfer ratio between the 4 groups.

Regarding maternal outcomes, 12 (7.2%) were hospitalized, 28 (16.9%) had a C-section and 137 (83%) had a vaginal delivery. Neonatal outcomes were favorable with only 14 cases (8.4%) transferred to the neonatology intensive care unit (NICU) and 11 (6.6%) preterm births observed between 29 and 36 WG among which 5 were born after 34 WG. Finally, 165 newborns, only 3 (1.8%) had intra uterine growth restriction (IUGR). We did not observe a correlation between the gestational age at maternal infection and prematurity (*p* value = 0.35) or IUGR (*p* value = 0.56), Nevertheless, definite conclusions are difficult to draw due to the low number of IUGR and premature deliveries included, which might not allow to reach statistical significance.

## Discussion

In this study, the SARS-CoV-2 materno-fetal transmission rate was low {(0.63%) IC_95%_ [0.02%; 3.48%]}. On the opposite, we observed an efficient transplacental transfer of specific SARS CoV-2 IgG with an average placental transfer ratio of 1.27 (IC 95% [0.69–2.89]). The transfer ratio increased with increasing time between the onset of maternal infection and delivery (*P* Value < 0.001). Maternal transplacental antibody transfer did not differ significantly depending on maternal disease severity.

In most studies, the level of vertical transmission was low, estimated to range between 3.2% and 5%^25,33–36^. In the largest published meta-analysis including 936 patients from a set of 38 cohorts and case series, the level of vertical transmission reached 3.2% IC 95% [2.2–4.3%]^25^. However, because of a lack of systematic and standardized samples’ collection at delivery in included studies, definite conclusions are difficult to draw. The present study is a large, multicenter study, designed to obtain a systematic collection of dyad mothers-newborns samples at birth and further strengthen the low risk of placental SARS-CoV-2 transmission, with a rate of less 1% in our study^25,33–36^.

Evidence of efficient placental SARS-CoV-2 antibodies transfer had been previously demonstrated^25,28^, but uncertainties remained regarding the impact of maternal disease severity and the delay required to obtain efficient transfer. Similarly to others, we observed that the placental antibody transfer was proportional to time elapsed since maternal infection^27–31,36–38^. Indeed, Brebant et al*.*^30^ showed that a transfer ratio above 1 was positively associated with a longer delay from maternal positive SARS-CoV-2 RT-PCR to delivery. Similarly, recent data from Song et al*.*^28^ demonstrated than the transfer ratio of IgG was higher when the first maternal positive RT-PCR occurred 60–180 days before the delivery compared with < 60 days (1.2 vs. 0.6, *p* < 0.0001). Interestingly, we did not observe a correlation between maternal disease severity and placental antibody transfer ratio. According to Trinité et al*.*^39^, hospitalized individuals develop higher titers compared to mild-symptomatic and asymptomatic individuals, which would suggest a more efficient placental antibody transfer among severely ill mothers. In accordance, Song et al*.*^28^ demonstrated that the transfer ratio was significantly higher in the mothers who were severely to critically symptomatic (1.6, 95% CI 1.42–2.49, n = 4) compared to mothers who were asymptomatic (1.0, 95% CI 0.62–1.14, n = 23) (1.6 vs. 1.0, *p *= 0.003) or mildly symptomatic (0.9, 95% CI 0.81–1.09, n = 50) (1.6 vs. 0.9, *p *= 0.002). The low number of severe cases included in our cohort might explain the absence of association observed in our study.

Our study represents one of the largest cohorts of maternal SARS-CoV-2 infection with a systematic description of fetal/neonatal outcomes. Interestingly, we observed a low rate of prematurity and IUGR compared to previous studies^15,21,33,40–44^. Similarly, we did not observe an increased risk of C-Sect.^15,16,45,46^. Nevertheless, our study was not designed to explore the risks of prematurity or IUGR in case of maternal infection and the numbers, especially of severe cases, are too small to draw any conclusion.

Strengths of our study include the prospective inclusion of patients throughout the pregnancy, allowing a representation of the three trimesters of the pregnancy, as well as a large cohort of patients (165 mother-infant dyads) including both symptomatic and asymptomatic form of SARS-CoV-2 infection. Furthermore, the systematic collection of the newborns’ samples immediately after birth limits the risk of contamination and interpretation bias.

A limitation of our study might be the possible underestimation of materno-fetal SARS-CoV-2 transmission due to the use of a restrictive definition of confirmed congenital infection. Our study was carried out in 2020, prior to the publication of the WHO classification of materno-fetal transmission^47^. By opposition to the WHO classification, which also includes neonatal positive samples for SARS-CoV-2 taken within the first 24 h of life to confirm a congenital infection, we only considered samples taken immediately at birth, as suggested by Shah et al*..* Nevertheless, all newborns were hospitalized for a minimum of 48 h after birth and severe adverse outcomes would have been recorded, suggesting that even in the case of missed congenital infection, the outcome would have be favorable. Another limitation is the absence of analysis carried out on patients with adverse outcomes like stillbirths or miscarriages. Miscarriage tissues were not analyzed in our study and fetal cord sera in case of stillbirth were, unfortunately, not available for analysis because fetal blood was clotted at the time of collection. Histopathological analysis of the placenta was not performed. Although we cannot exclude that some cases of stillbirths/ miscarriages were related to a congenital SARS CoV-2 infection, the numbers in our cohort were low (n = 6), suggesting that this would not significantly influence our conclusions. Finally, our reported rate of congenital SARS-CoV-2 infection may not reflect all cases of placental transmission, as a progressive clearance of the virus by placenta may occur during pregnancy which would impair its detection at birth^48^.

We chose to include both women with positive RT-PCR for SARS-CoV-2 and women with a negative PCR test but a positive serology for SARS-Cov-2 and compatible symptoms. Although we cannot exclude a memory bias and a potential infection before the inclusion, the presence of compatible symptoms during the current pregnancy lowers the risk.

The population included was an unvaccinated population allowing the evaluation of the transfer ratio following maternal infection. According to Beharier et al*.*^49^, the transfer ratio of IgG from a vaccinated population of pregnant women is similar to the one from an infected population. Similarly to what we observed in a natural infection, Atyeo et al*.*^50^ demonstrated a higher transfer efficiency following first trimester vaccination. Others have even demonstrated efficient antibody transfer, even when the vaccination took place prior to conception^51–53^. Considering these results, effective antibody transfer following maternal vaccination might be higher during the first part of the pregnancy and vaccination campaigns should target the first and second trimester of the pregnancy^54^. Some studies have raised the possibility of increased passive neonatal immunity when mothers have both been vaccinated and infected during pregnancy; adjunctive vaccination following maternal infection needs to be further explored^55^.

## Conclusions

In this large multicenter prospective study, we demonstrated a low rate of materno-fetal transmission. The placental IgG antibody transfer from the mother to the fetus seems to be higher when SARS-CoV-2 infection occurs during the first and second trimesters. These findings support SARS-CoV-2 vaccination of pregnant women during the first and the second trimester to ensure efficient neonatal protection through passive immunity.

## Data Availability

The data used and/or analyzed during the current study are available from the corresponding author upon reasonable request.
